# Principle and Application of Frequency-Domain Characteristic Analysis of Fractional-Order Memristor

**DOI:** 10.3390/mi13091512

**Published:** 2022-09-12

**Authors:** Bo Yu, Yifei Pu, Qiuyan He, Xiao Yuan

**Affiliations:** 1 College of Computer Science, Sichuan University, Chengdu 610065, China; 2 School of Physics and Engineering Technology, Chengdu Normal University, Chengdu 611130, China; 3 College of Electronics and Information Engineering, Sichuan University, Chengdu 610065, China

**Keywords:** fractional-order circuits/systems, mem-element, memristor, memcapacitor, meminductor

## Abstract

Scaling fractional-order memristor circuit is important for realizing a fractional-order memristor. However, the effective operating-frequency range, operation order, and fractional-order memristance of the scaling fractional-order memristor circuit have not been studied thoroughly; that is, the fractional-order memristance in the effective operating-frequency range has not been calculated quantitatively. The fractional-order memristance is a similar and equally important concept as memristance, memcapacitance, and meminductance. In this paper, the frequency-domain characteristic-analysis principle of the fractional-order memristor is proposed based on the order- and F-frequency characteristic functions. The reasons for selecting the order- and F-frequency characteristic functions are explained. Subsequently, the correctness of the frequency-domain characteristic analysis using the order- and F-frequency characteristic functions is verified from multiple perspectives. Finally, the principle of the frequency-domain characteristic analysis is applied to the recently realized chain-scaling fractional-order memristor circuit. The results of this study indicate that the principle of the frequency-domain characteristic analysis of the fractional-order memristor can successfully calculate the fractional-order memristance of the chain-scaling fractional-order memristor circuit. The proposed principle of frequency-domain characteristic analysis can also be applied to mem-elements, such as memristors, memcapacitors, and meminductors. The main contribution of this study is the principle of the frequency-domain characteristic analysis of the fractional-order memristor based on the order- and F-frequency characteristic functions.

## 1. Introduction

A memristor, proposed by Chua in 1971 [1], is a type of missing-circuit element. The memristor is used to establish a constitutive relationship between charge and flux. It is a nonlinear circuit element whose memristance changes according to the history of the input current or voltage. Although there are broader classes of physical devices and systems that have memristor-like properties, they cannot be used for modeling when using the memristor to model physical devices and systems. Therefore, Chua extended the application of the memristor to memristive systems [2]. In 2008, HP Labs produced the first memristor device [3]. The memristor has increasingly attracted research attention and has been widely used [4,5,6,7,8,9,10]. Based on the concept of the memristor, a memcapacitor and meminductor have also been proposed. The two constitutive variables of these mem-elements show pinched hysteretic loops [11]. Further, these mem-elements provide more choice for circuit and system design. Moreover, they promote the realization of concepts such as a nonvolatile trigger and parallel in-memory multiply-accumulate operations [4,5,6,7]. Research on mem-elements includes theoretical analysis [2,12,13,14], emulator design [15,16,17,18], device implementation [19,20,21], and application system design [4,5,6,7,8,9,10,22,23,24,25,26].

According to the fractional calculus theory [27], the operation orders of the memristor, memcapacitor, and meminductor are 0, −1, and +1, respectively. Pu and Yuan proposed the concept of a fractional-order memristor to obtain mem-elements with a fractional-order [28]. The units and dimensions of the fractional-order memristance are the same as those of a fractance. Fracmemristor and fracmemristance are portmanteaus for fractional-order memristor and memristance, respectively [28]. Pu et al. were the first to use the fracmemristor for designing an intelligent prediction model in the field of the financial technology; they developed a string-scaling fracmemristor circuit [29]. Further, Pu et al. designed a novel circuit for a fractional-order memristive neural synaptic weighting using a fracmemristor [30].

The fracmemristor is a new concept, and there has been a lack of commercially available fracmemristor devices. Currently, memristor emulators are used in place of the resistors in scaling factor circuits for realizing fracmemristor circuits having suitable electrical characteristics. The implemented fracmemristor circuits have lattice scaling [31], ladder scaling [32], chain scaling [33], and other configurations, which are collectively referred to as scaling fracmemristor circuits. Such a fracmemristor is the focus of this study. There is another type of fracmemristor, whose units and dimensions are the same as those of the memristor and wherein the internal state variables are the fractional integral of the voltage or current [34,35,36].

Important parameters of the fracmemristor include the fracmemristance and operational order [31]. The fracmemristance is a similar and equally important concept as memristance, memcapacitance, and meminductance. The scaling fracmemristor circuit is important for implementing a fracmemristor circuit [31,32,33]. The effective operating-frequency range is an important index of the scaling fracmemristor circuit [32]. The scaling fracmemristor circuit must meet not only the operational order requirements of the fracmemristor in the effective operating-frequency range but also the requirements of the change in fracmemristance. The fracmemristance in the effective operating-frequency range is constant when the internal state variable is fixed. It should also change when the internal state variable changes. However, the quantification of the numerical variation of the fracmemristance in the effective operating-frequency range is yet to be solved [31,32,33]. This is because the impedance function of the scaling fracmemristor circuit is an irregular iterative scaling equation, the analytical solution of which remains a challenging problem [31,32,33,37,38].

The theory of the fractor circuit is the basis of implementing the fracmemristor circuit [31,32,33,37,38]. Operation order and fractance are two important parameters of the fractor [37,38]. Yuan proposed an order-frequency characteristic function to quantify the operational order [38], whereas Yu et al. proposed the F-frequency characteristic function to quantify the fractance [39], of the fractor circuit in the frequency domain. The order- and F-frequency characteristic functions have been widely used in the fractor circuit [37,38,39,40,41,42,43,44]. Pu et al. first used the order-frequency characteristic function to obtain the effective operating-frequency range of the scaling fracmemristor circuit [32].

Small-signal analyses are important methods for analyzing the memristor and its circuit [2,12,13,14]. Chua presented the small-signal equivalent circuit of a memristive system to distinguish a memristive device from other systems [2]. Liang et al. used the small-signal analysis to assess the importance of the DC V–I characteristics in the performance of a locally active memristor [14]. According to the principle of small-signal analysis, the small-signal equivalent circuit of the scaling fracmemristor circuit is the scaling fractor circuit, and the small-signal impedance function of the scaling fracmemristor impedance function is the impedance function of the scaling fractor circuit. The order- and F-frequency characteristic functions are effectively used in the frequency-domain analysis of the operational order and fractance of the scaling factor circuit [37,38,39,40,41,42,43,44]. An effective operating-frequency range, operational order, and fracmemristance of the fracmemristor circuit in the frequency domain can be obtained by applying the order- and F-frequency characteristic functions to the small-signal equivalent circuit and impedance function of the fracmemristor circuit.

Given this context, this study introduced the order- and F-frequency characteristic functions for obtaining the frequency-domain characteristic-analysis principle of the fracmemristor to calculate the fracmemristance of the scaling fracmemristor circuit. The main contributions of this study are as follows:

- The frequency-domain characteristic-analysis principle of the fracmemristor can be used to numerically calculate the effective operating-frequency range and frequency-domain approximation performance of the fracmemristor circuit.
- The principle can help calculate the fracmemristance of the scaling fracmemristor circuit.
- The half-order chain-type fracmemristor circuit, which is beneficial for verifying the correctness of the frequency-domain characteristic-analysis principle of the fracmemristor more scientifically as compared with the scaling fracmemristor circuit, is proposed.
- The fracmemristor acts as a memcapacitor, memristor, and meminductor when its operation orders are extended to −1, 0, and +1, respectively. The principle of the frequency-domain characteristic analysis of the fracmemristor can also be used for mem-elements, which are widely used in memristors, memcapacitors, and meminductors.

The remainder of this manuscript is organized as follows. The basic concepts of the fracmemristor and the research problems considered this study are introduced in Section 2. The reason for choosing the order- and F-frequency characteristic functions is clarified in Section 3. Further, the principle of the frequency-domain characteristic analysis of fracmemristor is presented. In Section 4, the principle of the frequency-domain characteristic analysis is applied to the scaling fracmemristor circuit, the approximation performance of the scaling fracmemristor circuit is obtained, and the fracmemristance of the scaling fracmemristor circuit is solved. In Section 5, the theory of the frequency-domain characteristic analysis is applied to the memristor, memcapacitor, and meminductor to demonstrate its wide applicability. Finally, Section 6 presents the conclusion of the study.

## 2. Preliminaries

In this section, few basic concepts of fracmemristor (e.g., impedance function and fracmemristance) are introduced. Subsequently, the concept of the fracmemristor circuit is discussed, and basic knowledge about the scaling fracmemristor circuit is summarized. Finally, the research problems to be solved in this study are presented in detail.

The fracmemristor is a two-terminal circuit element that includes capacitive and inductive fracmemristors. Pu et al. derived the driving-point impedance function of the arbitrary operation-order fracmemristor in its natural realization form as [28,31]

(1) $$FM_{\mu }=\left\{\begin{array}{c} FM_{\mu }^{c}=FM_{-(\eta +p)}^{c}=c^{\mu }{\left[R\left(s\right)\right]}^{1-p}s^{\mu }\left(\mu <0\right)\\ FM_{\mu }^{l}=FM_{\eta +p}^{l}=l^{\mu }{\left[R\left(s\right)\right]}^{1-p}s^{\mu }\left(\mu >0\right) \end{array}\right.,$$

where $FM_{\mu }^{c}$, $FM_{\mu }^{l}$, *c*, *l*, *s*, μ, and η+p represent the impedance of the ideal capacitive fracmemristor, impedance of the ideal inductive fracmemristor, capacitance, inductance, complex variable of the Laplace transform, operational order of the fracmemristor, and non-negative real number, respectively. Further, η represents a non-negative integer and 0≤p≤1. $\eta =\left\lfloor \left|\mu \right|\right\rfloor$, ⌊⌋ represents the round towards minus infinity.

The fracmemristance of the fracmemristor is given by

(2) $$m_{\mu }\left(x\right)=\left\{\begin{array}{c} m_{\mu }^{c}\left(x\right)=c^{\mu }L^{-1}\left\{{\left[R\left(s\right)\right]}^{1-p}\right\}\left(\mu <0\right)\\ m_{\mu }^{l}\left(x\right)=l^{\mu }L^{-1}\left\{{\left[R\left(s\right)\right]}^{1-p}\right\}\left(\mu >0\right) \end{array}\right.,$$

where $m_{\mu }^{c}\left(x\right)$, $m_{\mu }^{l}\left(x\right)$, and $L^{-1}\left\{\right\}$ represent the capacitive fracmemristance, inductive fracmemristance, and inverse Laplace transform, respectively. Further, *x* represents the internal state variable. Equation (1) represents the impedance function of charge-controlled fracmemristor, and Equation (2) represents the charge-controlled fracmemristance when *x* represents the charge *q*. Equation (1) represents the impedance function of the flux-controlled fracmemristor and Equation (2) represents the flux-controlled fracmemristance when *x* represents the flux φ (the integral value of the voltage). The unit and dimension of the fracmemristance are the same as those of the fractance. Further, *x* represents a variety of other variables such as the fractional-order integral of the voltage and the fractional-order integral of the current. The capacitive fracmemristance $m_{\mu }^{c}$, inductive fracmemristance $m_{\mu }^{l}$, and internal state variable *x* are related to other physical quantities such as voltage *u* and current *i*; therefore, the corresponding function of the fracmemristance includes $m_{\mu }^{c}\left(x,u\right)$, $m_{\mu }^{c}\left(x,i\right)$, $m_{\mu }^{l}\left(x,u\right)$, and $m_{\mu }^{l}\left(x,i\right)$.

Equations (1) and (2) can not only be used for the fracmemristor; they also represent the impedance of more elements and their parameter values based on the different values of their parameters. Equation (1) denotes the impedance function of the memristor and Equation (2) denotes the memristance when μ=0, η=0, and p=0. The impedance function of the memristor is $FM_{0}=R\left(s\right)$ and the memristance $m_{0}\left(x\right)=L^{-1}\left[R\left(s\right)\right]$. Equation (1) represents the impedance function of the memcapacitor, and Equation (2) denotes the memcapacitance when μ=−1, η=1, and p=0. The impedance function of the memcapacitor is $FM_{-1}^{c}=c^{-1}R\left(s\right)s^{-1}$; the lumped parameter value of the memcapacitor is $m_{-1}^{c}\left(x\right)=c^{-1}L^{-1}\left\{R(s)\right\}$. The lumped parameter value of the memcapacitor is the inverse of the memcapacitance. Equation (1) denotes the impedance function of the meminductor and Equation (2) denotes the meminductance when μ=+1, η=1, and p=0. The impedance function of the meminductor is $FM_{+1}^{l}=lR\left(s\right)s$; the meminductance is $m_{+1}^{l}\left(x\right)=lL^{-1}\left\{R(s)\right\}$. Equations (1) and (2) can represent the impedance function of elements such as the resistor, capacitor, inductor, and their lumped parameter values when R(s) is constant. For example, when R(s)=R=r and μ=0, we have η=0 and p=0, where (1) denotes the impedance function of the resistor and (2) denotes the resistance. The impedance function of the resistor is $FM_{0}=R$, and the resistance $m_{0}=r$. The types of elements that can be represented by Equations (1) and (2) are presented in Table 1.

Therefore, researchers have proposed a variety of fracmemristor circuits to study the theory and application of the fracmemristor [31,32,33]. The electrical characteristics of fracmemristor are approximately realized under an effective operating-frequency range and acceptable accuracy. In terms of physics, the active two-terminal circuit network that can be realized by the circuit is used for investigating the electrical and operational characteristics of the fracmemristor. In terms of mathematics, a circuit-realizable impedance-function approximation (1) as shown in the impedance is constructed.

The fracmemristance and operational order can be solved by the impedance function $\overset{-}{Z}\left(s\right)$ of the scaling fracmemristor circuit. Further, $\overset{-}{Z}\left(s\right)$ should be calculated from the scaling iteration formula; the corresponding irregular iterative scaling equation is [31,32,33] given as

(3) $$Z\left(s\right)=F(\alpha \overset{-}{Z}\left(\alpha \beta s\right)),$$

where α and β are the progression ratio of the reference memristance and reference capacitance, respectively. α and β are positive real numbers, and α≠1 and β≠1. The iterative scaling equation analytic solution is a challenging mathematical problem [31,32,33,37,38]. The operational order of the scaling fracmemristor circuit is obtained using an approximate solution [31,32,33]. The operation order of the scaling fractional memristor is [31,32,33]

(4) μ≈−lgα/lg(αβ).

The fracmemristor circuit of arbitrary fractional operation order can be obtained by adjusting the values of α and β. The operation order μ is an approximate value, and its error must be quantified and analyzed. The analytical solution of the fracmemristance including the approximate solution remains an unsolved problem.

The electrical characteristics of the fracmemristor can be realized in the effective operating-frequency range of the scaling fracmemristor circuit. Pu et al. were the first to obtain the effective operating-frequency range of the scaling fracmemristor circuit using the order-frequency characteristic curve [32]. An important property of the fracmemristor is that the fracmemristance varies according to a change in the state variable *x*. The fracmemristance $m_{\mu }\left(x\right)$ in the effective operating-frequency range is constant when the state variable *x* fixed. The scaling fracmemristor circuit not only fulfills the operational order requirements, but it also satisfies the requirements of the change in the fracmemristance $m_{\mu }\left(x\right)$. However, only the operational order μ within the effective operating-frequency range can be obtained using the order-frequency characteristic. The variation of fracmemristance in the effective operating-frequency range is yet to be confirmed. Further, the fracmemristance of the scaling fracmemristor circuit has not been solved theoretically. With the further study of the fracmemristor, it is necessary to quantify the fracmemristance and the approximate accuracy of the operational order in the frequency domain.

To solve the aforementioned problems, this study introduced the order- and F-frequency characteristic functions to obtain the frequency-domain characteristic-analysis principle of the fracmemristor.

## 3. Frequency-Domain Characteristic-Analysis Principle of the Fracmemristor

In this section, the frequency-domain characteristic-analysis principle of the circuit elements is introduced. This is followed by the explanation for choosing the order- and F-frequency characteristic functions to obtain the frequency-domain characteristic-analysis principle of the fracmemristor. Subsequently, the impedance function of the ideal fracmemristor and small-signal analysis method are used to verify the accuracy of the frequency-domain characteristic analysis of the fracmemristor using the order- and F-frequency characteristic functions. Finally, a half-order chain-type fracmemristor circuit is proposed to verify the principle of the frequency-domain characteristic analysis. The frequency-domain characteristics of the small-signal equivalent circuit of the half-order chain-type fracmemristor circuit are analyzed theoretically. The results of the analysis not only proves the principle of the frequency-domain characteristic analysis of fracmemristor but also verifies its accuracy from a circuit perspective.

### 3.1. Frequency-Domain Characteristic-Analysis Principle of Circuit Elements

If the impedance function of the linear circuit element is Z(s), the variable *s* is replaced by the frequency index variable ϖ [37]. That is,

(5) $$s=j2\pi 10^{\varpi }.$$

Thus, the amplitude–frequency characteristic function of Z(s) is obtained as

(6) $$\Lambda \left(\varpi \right)=\mathrm{lg}\left|Z\left(j2\pi 10^{\varpi }\right)\right|,$$

and the phase–frequency characteristic function as

(7) $$\theta \left(\varpi \right)=\arg\left\{Z\left(j2\pi 10^{\varpi }\right)\right\}.$$

The amplitude–frequency characteristic function Λ(ϖ) denotes the peak-to-peak ratio of the sinusoidal voltage signal at the terminal of the element to the corresponding sinusoidal current signal. The phase–frequency characteristic function θ(ϖ) denotes the phase difference between the sinusoidal voltage signal at the terminal of the element and the corresponding sinusoidal current signal.

The order-frequency characteristic function can be used not only for researching the fractor circuit but also for the quantitative analysis of the circuit elements, linear circuits, and systems in the frequency domain. The order-frequency characteristic function of Z(s) is [38]

(8) $$O\left(\varpi \right)=\frac{d\Lambda (\varpi )}{d\varpi }=\frac{\mathrm{lg}\left|Z\left(j2\pi 10^{\varpi }\right)\right|}{d\varpi }.$$

The F-frequency characteristic function of Z(s) is [39]

(9) $$\Gamma \left(\varpi \right)=\mathrm{lg}F\left(\varpi \right)=\Lambda \left(\varpi \right)-O\left(\varpi \right)\left[\varpi +\mathrm{lg}\left(2\pi \right)\right]=\mathrm{lg}\left|Z\left(j2\pi 10^{\varpi }\right)\right|-O\left(\varpi \right)\left[\varpi +\mathrm{lg}\left(2\pi \right)\right].$$

The F-frequency characteristic function can also be used in the frequency-domain quantitative analysis of circuit elements, linear circuits, and systems.

The order-frequency characteristic functions of the ideal resistor, capacitor, and inductor are O(ϖ)=0, O(ϖ)=−1, and O(ϖ)=+1 respectively; further, the F-frequency characteristic functions F(ϖ) indicate its resistance, inverse of the capacitance, and inductance, respectively.

The amplitude–frequency and phase–frequency characteristic functions are especially important frequency-characteristic functions in circuits and systems [45]. The amplitude–frequency and phase–frequency characteristics are used in the analysis of linear systems. Mem-elements belong to the category of nonlinear systems. The small-signal analysis method is required for using the amplitude–frequency and phase–frequency characteristics functions in the analysis of mem-elements [2,12,13,14]. According to Equations (8) and (9), both the order- and F-frequency characteristic functions are contained in the amplitude-frequency characteristic function.

### 3.2. Order-Frequency and F-Frequency Characteristics of the Ideal Fracmemristor Impedance Function

The small-signal impedance function of the ideal fracmemristor is substituted into the order- and F-frequency characteristic functions; the accuracy of the application of the order- and F-frequency characteristic functions to the frequency-domain characteristic analysis of the fracmemristor is verified. According to Equation (2), the fracmemristance is controlled by the state variable *x*, which results in the impedance function $FM_{\mu }$ of the ideal fracmemristor as a nonlinear function. Assuming that the operating point of ideal fracmemristor is $Q\left(U_{0},I_{0}\right)$ and the corresponding state variable $x=X_{0}$, R(s)=r in Equations (1) and (2). The small-signal impedance function can be represented by $Z\left(s,Q\right)=FM_{\mu }{\left(s,Q\right)|}_{R(s)=r}$. According to Equation (8), the order-frequency characteristic function value can be obtained as

(10) $$O\left(\varpi ,Q\right)=\frac{\mathrm{lg}\left|FM_{\mu }\left(j2\pi 10^{\varpi },Q\right)\right|}{d\varpi }=\mu .$$

Based on Equation (9), the F-frequency characteristic function value can be obtained as

(11) $$\Gamma \left(\varpi ,Q\right)=\mathrm{lg}F\left(\varpi ,Q\right)=\mathrm{lg}\left|FM_{\mu }\left(j2\pi 10^{\varpi },Q\right)\right|-O\left(\varpi ,Q\right)\left[\varpi +\mathrm{lg}\left(2\pi \right)\right]=\mathrm{lg}m_{\mu }.$$

According to Equations (10) and (11), the order-frequency characteristic function value is equal to the operation order of the fracmemristor, and the F-frequency characteristic function value F(ϖ,Q) is equal to the fracmemristance $m_{\mu }$. Thus, it is theoretically proved that the order- and F-frequency characteristic functions are suitable for the frequency-domain analysis of the fracmemristor.

### 3.3. Frequency-Domain Characteristic Analysis of the Half-Order Chain-Type Fracmemristor Circuit

The fracmemristance is yet to be solved for all implemented scaling fracmemristor circuits [31,32,33]. The half-order chain-type fracmemristor circuit is proposed by replacing the resistor with the memristor in the half-order chain-type fractor circuit [38]. The fracmemristance of the half-order chain-type fracmemristor circuit can be solved analytically to compare and verify the accuracy of the F-frequency characteristic function.

Further, the frequency-domain characteristics of the half-order chain-type fracmemristor circuit are analyzed. The configuration of the half-order chain-type fracmemristor circuit is provided; using circuit theory, the operation order and F characteristic value of circuit in a different frequency range are analyzed. Subsequently, the impedance function of the half-order chain-type fracmemristor circuit is substituted into the order- and F-frequency characteristic functions; the operation order and F characteristic value of the circuit in a different frequency range are calculated theoretically. The theory confirms that the order- and F-frequency characteristic functions are suitable for the frequency-domain characteristic analysis of the fracmemristor circuit. Finally, the frequency characteristic analysis theory of the fracmemristor circuit is verified by investigating the curves of the order- and F-frequency characteristic functions.

#### 3.3.1. Half-Order Chain-Type Fracmemristor Circuit

**Figure 1:**
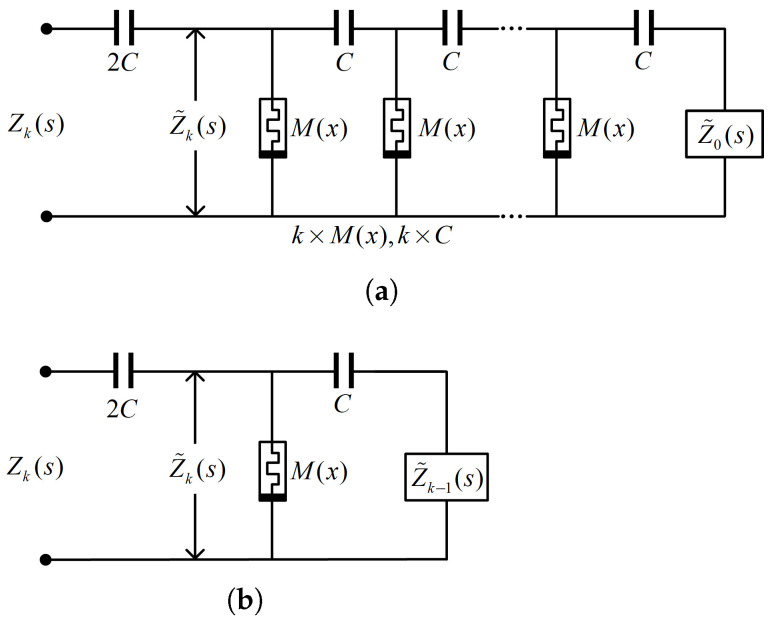
Half-order chain-type fracmemristor circuit: (**a**) circuit configuration; (**b**) iterating circuit.

The configuration of the half-order chain-type fracmemristor circuit is shown in Figure 1a. Figure 1b shows its iterating circuit. M(x), *C*, and *k* denote the reference memristance, reference capacitance, and total number of subcircuits, respectively. The state variable *x* is controlled by the terminal voltage or current. The half-order chain-type fracmemristor circuit has an ideal approximation property in the effective operating-frequency range. $Z_{0}\left(s\right)$ represents the initial impedance, $Z_{0}\left(s\right)=M\left(x\right)$.

According to the iterative circuit shown in Figure 1b, the input impedance ${\tilde{Z}}_{k}\left(s\right)$ is described by the iterative algorithm formula

(12) $${\tilde{Z}}_{k}\left(s\right)=\frac{M\left(x\right)\left[1+sC{\tilde{Z}}_{k-1}\left(s\right)\right]}{1+sC{\tilde{Z}}_{k-1}\left(s\right)+sCM\left(x\right)}.$$

When k→∞, the limit impedance calculated from Equation (12) is

(13) $$\tilde{Z}\left(s\right)=\frac{1}{2sC}\left(\sqrt{1+\frac{4s}{\Omega_{1}}}-1\right).$$

According to Figure 1b, the input impedance is $Z_{k}\left(s\right)={\tilde{Z}}_{k}\left(s\right)+\frac{1}{2sC}$, and therefore, the limit impedance can be obtained as

(14) $$Z\left(s\right)=\frac{1}{2sC}\sqrt{1+\frac{4s}{\Omega_{1}}},$$

where $\Omega_{1}=\frac{1}{M(x)C}$ represents the eigen angular frequency.

Assume k→∞ for the half-order chain-type fracmemristor circuit. When $\left|s\right|\gg \Omega_{1}$ (the effective operating-frequency range), $\frac{4s}{\Omega_{1}}\gg 1$. Using Equation (14), $Z\left(s\right)=\sqrt{\frac{M(x)}{C}}s^{-0.5}$. Fracmemristance $m_{\mu }^{c}\left(x\right)=\sqrt{\frac{M(x)}{C}}$ by implementing the operation order of the μ=−0.5 fracmemristor.

The real circuit has a finite value of *k*. The operation characteristics of the signal frequencies at different values are considered to discuss the frequency-domain characteristics of the half-order chain-type fracmemristor circuit with a finite value of *k*.

(1) In the range of the angular frequency $\left[\Omega_{1},\Omega_{k}\right]$, the half-order chain-type fracmemristor circuit with a finite *k* realizes the operation characteristic of the half-order fracmemristor. When $\Omega_{1}<\left|s\right|<\Omega_{k}$ [38], $Z\left(s\right)\approx \sqrt{\frac{M(x)}{C}}s^{-0.5}$, implements the operations order μ=−0.5 and fracmemristance $m_{-0.5}^{c}\left(x\right)=\sqrt{\frac{M(x)}{C}}$. In this scenario, the order-frequency characteristic function O(s)=−0.5 and the F-frequency characteristic function $F\left(s\right)=\sqrt{\frac{M(x)}{C}}$.

(2) When the angular frequency has the maximum value, i.e., when it approaches infinity, the half-order chain-type fracmemristor circuit with finite *k* realizes the operation characteristic of the memristor. The capacitance $\left|\frac{1}{sC}\right|\to 0$ when $\left|s\right|\to \infty$. The half-order chain-type fracmemristor circuit with finite *k* is equivalent to k+1 memristor M(x) in parallel. $Z\left(s\right)=\frac{M(x)}{k+1}$, implements the operations order μ=0 and the memristance for $m_{0}^{c}\left(x\right)=\frac{M(x)}{k+1}$ of the zero-order memristor. In this scenario, the order-frequency characteristic function O(s)=0, and the F-frequency characteristic function $F\left(s\right)=\frac{M(x)}{k+1}$.

(3) When the angular frequency is extremely low, i.e., when it approaches zero, the half-order chain-type fracmemristor circuit with finite *k* realizes the operation characteristic of the capacitor. When $\left|s\right|\to 0$, the capacitance impedance $\left|\frac{1}{sC}\right|\gg M\left(x\right)$. The half-order chain-type fracmemristor circuit with finite *k* is equivalent to the capacitor with capacitance 2C. $Z\left(s\right)=\frac{1}{2sC}$, implements the operations order μ=−1 and the capacitance 2C of capacitor. In this situation, the order-frequency characteristic function O(s)=−1, and the F-frequency characteristic function $F\left(s\right)=\frac{1}{2C}$.

(4) When the angular frequency is higher than $\Omega_{k}$, the half-order chain-type fracmemristor circuit with finite *k* is realized from the half-order fracmemristor to the memristor with an increase in angular frequency. When $\Omega_{k}<\left|s\right|<\infty$, $\sqrt{\frac{M(x)}{C}}s^{-0.5}>Z\left(s\right)>\frac{M(x)}{k+1}$, implements the μ=−0.5 and $m_{-0.5}^{c}\left(x\right)=\sqrt{\frac{M(x)}{C}}$ fracmemristor to the μ=0 and $m_{0}^{c}\left(x\right)=\frac{M(x)}{k+1}$ memristor change processes. The order-frequency characteristic function O(s) changes from −0.5 to 0 and the F-frequency characteristic function F(s) changes from $\sqrt{\frac{M(x)}{C}}$ to $\frac{M(x)}{k+1}$ with an increase in the angular frequency.

(5) When the angular frequency is less than $\Omega_{1}$, the half-order chain-type fracmemristor circuit with finite *k* is realized from the half-order fracmemristor to the capacitor with an increase in the angular frequency. When $\Omega_{1}>\left|s\right|>0$, $\sqrt{\frac{M(x)}{C}}s^{-0.5}<Z\left(s\right)<\frac{1}{2sC}$, implements the μ=−0.5 and $m_{-0.5}^{c}\left(x\right)=\sqrt{\frac{M(x)}{C}}$ fracmemristors to the μ=−1 and $m_{-1}^{c}=\frac{1}{2C}$ capacitor change processes. With a decrease in the angular frequency, the order-frequency characteristic function O(s) changes from −0.5 to −1 and the F-frequency characteristic function F(s) changes from $\sqrt{\frac{M(x)}{C}}$ to $\frac{1}{2C}$.

#### 3.3.2. Theoretical Verification of the Order-Frequency and F-Frequency Characteristics

The absolute value of the limiting impedance when k→∞ is as shown in Equation (14).

(15) $$\left|Z(s)\right|=\left|\frac{1}{2sC}\sqrt{1+\frac{4s}{\Omega_{1}}}\right|.$$

Substitute Equation (5) into Equation (15) to obtain

(16) $$\left|Z(j2\pi 10^{\varpi })\right|=\left|\frac{1}{j4\pi 10^{\varpi }C}\sqrt{1+\frac{j8\pi 10^{\varpi }}{\Omega_{1}}}\right|.$$

By setting the frequency index variable $\varpi_{1}=\mathrm{lg}\frac{\Omega_{1}}{2\pi }$, $\Omega_{1}=2\pi 10^{\varpi_{1}}$. Then,

(17) $$\left|Z(j2\pi 10^{\varpi })\right|=\left|\frac{1}{j4\pi 10^{\varpi }C}\sqrt{1+j4\cdot 10^{\left(\varpi -\varpi_{1}\right)}}\right|.$$

The order-frequency characteristic function of the half-order chain-type fracmemristor circuit is obtained by substituting Equation (17) into Equation (8) as

(18) $$O\left(\varpi \right)=\frac{\mathrm{lg}\left|\frac{1}{j4\pi 10^{\varpi }C}\sqrt{1+j4\cdot 10^{\left(\varpi -\varpi_{1}\right)}}\right|}{d\varpi }.$$

The F-frequency characteristic function of the half-order chain-type fracmemristor circuit is obtained by substituting Equation (17) into Equation (9) as

(19) $$\Gamma \left(\varpi \right)=\mathrm{lg}\left|\frac{1}{j4\pi 10^{\varpi }C}\sqrt{1+j4\cdot 10^{\left(\varpi -\varpi_{1}\right)}}\right|-O\left(\varpi \right)\left[\varpi +\mathrm{lg}\left(2\pi \right)\right].$$

The half-order chain-type fracmemristor circuit with k→∞ is a high-frequency effective fracmemristor. $j4\cdot 10^{\left(\varpi -\varpi_{1}\right)}\gg 1$ when $\varpi \gg \varpi_{1}$ (the effective operating-frequency range). According to Equation (18), the order-frequency characteristics of the half-order chain-type fracmemristor circuit is

(20) $$O\left(\varpi \right)=\frac{\mathrm{lg}\left|\frac{1}{j4\pi 10^{\varpi }C}\sqrt{j4\cdot 10^{\left(\varpi -\varpi_{1}\right)}}\right|}{d\varpi }=-0.5.$$

According to Equation (19), the F-frequency characteristic of the half-order chain-type fracmemristor circuit is

(21) $$\Gamma \left(\varpi \right)=\mathrm{lg}F\left(\varpi \right)=\mathrm{lg}\left|\frac{1}{j4\pi 10^{\varpi }C}\sqrt{j4\cdot 10^{\left(\varpi -\varpi_{1}\right)}}\right|+\frac{\varpi +\mathrm{lg}(2\pi )}{2}=\mathrm{lg}\sqrt{\frac{M(x)}{C}},$$

that is,

(22) $$F\left(\varpi \right)=\sqrt{\frac{M(x)}{C}}.$$

Equations (20)–(22) are the results obtained when k→∞. The actual circuit has a finite number of *k*. The operation characteristics of signal frequencies at different values are elaborated to comprehensively discuss the frequency-domain characteristics of the half-order chain-type fracmemristor circuit with finite *k* using the order- and F-frequency characteristic functions.

(1) When $\varpi_{1}<\varpi <\varpi_{k}$ [38]. The order-frequency characteristic function O(ϖ)≈−0.5 is calculated from Equation (18). The F-frequency characteristic function $F\left(\varpi \right)\approx \sqrt{\frac{M(x)}{C}}$ is calculated from Equation (19).

(2) When ϖ→∞, $Z\left(j2\pi 10^{\varpi }\right)=\frac{M(x)}{k+1}$. The order-frequency characteristic function O(ϖ)=0 and F-frequency characteristic function $F\left(\varpi \right)=\frac{M(x)}{k+1}$ are calculated from Equations (8) and (9), respectively.

(3) When ϖ→0, $Z\left(j2\pi 10^{\varpi }\right)=\frac{1}{j4\pi 10^{\varpi }C}$. The order-frequency characteristic function O(ϖ)=−1 and F-frequency characteristic function $F\left(\varpi \right)=\frac{1}{2C}$ are calculated from Equations (8) and (9), respectively.

(4) When $\varpi_{k}<\varpi <\infty$, $\sqrt{\frac{M(x)}{C}}{\left(j2\pi 10^{\varpi }\right)}^{-0.5}>Z\left(j2\pi 10^{\varpi }\right)>\frac{M(x)}{k+1}$. With an increase in frequency, the order-frequency characteristic function O(ϖ) changes from −0.5 to 0, and the F-frequency characteristic function F(ϖ) changes from $\sqrt{\frac{M(x)}{C}}$ to $\frac{M(x)}{k+1}$.

(5) When $\varpi_{1}>\varpi >0$, $\sqrt{\frac{M(x)}{C}}{\left(j2\pi 10^{\varpi }\right)}^{-0.5}<Z\left(j2\pi 10^{\varpi }\right)<\frac{1}{j4\pi 10^{\varpi }C}$. With a decrease in frequency, the order-frequency characteristic function O(ϖ) changes from −0.5 to −1, and the F-frequency characteristic function F(ϖ) changes from $\sqrt{\frac{M(x)}{C}}$ to $\frac{1}{2C}$.

The above analysis results are consistent with the analysis results in Section 3.3.1; this indicates that the order- and F-frequency characteristic functions are suitable for the frequency-domain characteristic analysis of the fracmemristor circuits.

#### 3.3.3. Curve Verification of the Order-Frequency and F-Frequency Characteristic Functions

The memristance M(x) in the half-order chain-type fracmemristor circuit changes with the state variable *x*. To once again verify that the order- and F-frequency characteristic function are suitable for the frequency-domain characteristic analysis of the fracmemristor circuit, the order- and F-frequency characteristic curves of the half-order chain-type fracmemristor circuit are illustrated when the state variable *x* has different values.

**Figure 2:**
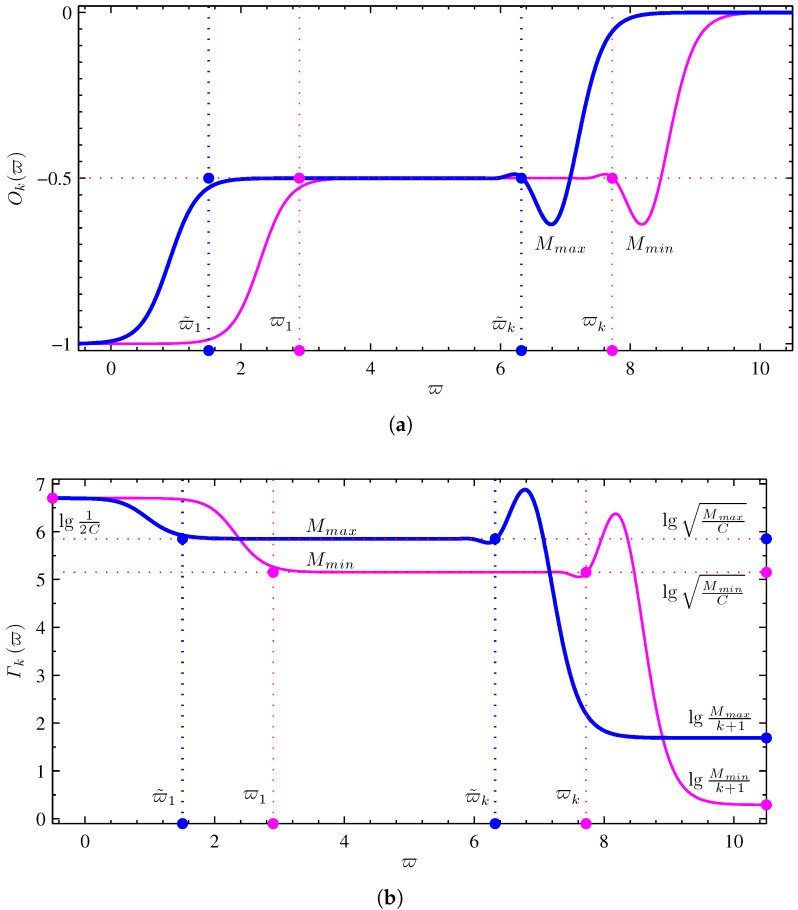
Frequency domain characteristic curve of the half-order chain-type fracmemristor circuit: (**a**) order-frequency characteristic curve; (**b**) F-frequency characteristic curve.

In the half-order chain-type fracmemristor circuit, the reference memristance varies with the input signal in the range of minimum and maximum values when considering k=1024 and capacitance C=0.1μF for the circuit shown in Figure 1a. Assuming that the operating point is $Q\left(U_{0},I_{0}\right)$, the corresponding state variable $x=X_{0}$. The impedance function $Z_{k}\left(j2\pi 10^{\varpi },Q\right)$ is obtained by substituting the parameters into Equation (12). The order- and F-frequency characteristic curves can be obtained by substituting impedance function $Z_{k}\left(j2\pi 10^{\varpi },Q\right)$ into Equations (8) and (9). $M_{max}$ and $M_{min}$ denote the maximum and minimum values of the reference memristance, respectively. Figure 2 shows the order- and F-frequency characteristic curves for $M\left(X_{0}\right)=M_{max}=50$ kΩ and $M\left(X_{0}\right)=M_{min}=2$ kΩ.

According to the characteristic curve of the order-frequency shown in Figure 2a, the half-order chain-type fracmemristor circuit can indeed achieve the operation of order μ=−0.5. When memristance $M\left(X_{0}\right)=M_{min}$, the effective operating-frequency range for realizing the operation order μ=−0.5 is $\left[\varpi_{1},\varpi_{k}\right]$. When memristance $M\left(X_{0}\right)=M_{max}$, the effective operating-frequency range for realizing the operation order is $\left[{\tilde{\varpi }}_{1},{\tilde{\varpi }}_{k}\right]$. The effective operating-frequency range for the operation order μ=−0.5 is changed with the memristance $M(X_{0})$, and the frequency range of the overlap $\left[\varpi_{1},{\tilde{\varpi }}_{k}\right]$ represents the effective operating-frequency range of the half-order chain-type fracmemristor circuit. When ϖ→0 and ϖ→∞, the order-frequency characteristic functions O(ϖ)=−1 and O(ϖ)=0, respectively. When $M\left(X_{0}\right)=M_{min}$ and $\varpi_{k}<\varpi <\infty$, the order-frequency characteristic function O(ϖ) changes from −0.5 to 0 with an increase in the frequency. When $M\left(X_{0}\right)=M_{min}$ and $\varpi_{1}>\varpi >0$, the order-frequency characteristic function O(ϖ) changes from −0.5 to −1 with a decrease in frequency. When $M\left(X_{0}\right)=M_{max}$ and ${\tilde{\varpi }}_{k}<\varpi <\infty$, the order-frequency characteristic function O(ϖ) changes from −0.5 to 0 with an increase in frequency. When $M\left(X_{0}\right)=M_{max}$ and ${\tilde{\varpi }}_{1}>\varpi >0$, the order-frequency characteristic function O(ϖ) changes from −0.5 to −1 with a decrease in frequency. The characteristic curve of order-frequency shown in Figure 2a is consistent with the analysis results presented in Section 3.3.1 and Section 3.3.2.

According to the F-frequency characteristic curve shown in Figure 2b, the half-order chain-type fracmemristor circuit realization of the fracmemristance $m_{-0.5}^{c}\left(X_{0}\right)=\sqrt{\frac{M(X_{0})}{C}}$ is the same as the effective operating-frequency range of operation order μ=−0.5. When ϖ→0, the F-frequency characteristic function $F\left(\varpi \right)=\frac{1}{2C}$. When $M\left(X_{0}\right)=M_{min}$ and $\varpi_{k}<\varpi <\infty$, the F-frequency characteristic function F(ϖ) changes from $\sqrt{\frac{M_{min}}{C}}$ to $\frac{M_{min}}{k+1}$ with an increase in frequency. When $M\left(X_{0}\right)=M_{min}$ and $\varpi_{1}>\varpi >0$, the F-frequency characteristic function F(ϖ) changes from $\sqrt{\frac{M_{min}}{C}}$ to $\frac{1}{2C}$ with a decrease in frequency. When $M\left(X_{0}\right)=M_{max}$ and ${\tilde{\varpi }}_{k}<\varpi <\infty$, the F-frequency characteristic function F(ϖ) changes from $\sqrt{\frac{M_{max}}{C}}$ to $\frac{M_{max}}{k+1}$ with an increase in frequency. When $M\left(X_{0}\right)=M_{max}$ and ${\tilde{\varpi }}_{1}>\varpi >0$, the F-frequency characteristic function F(ϖ) changes from $\sqrt{\frac{M_{max}}{C}}$ to $\frac{1}{2C}$ with a decrease in frequency. The characteristic curve of the F-frequency shown in Figure 2b is consistent with the analysis results presented in Section 3.3.1 and Section 3.3.2.

## 4. Frequency-Domain Characteristic Analysis of Scaling Fracmemristor Circuit

The implemented scaling fracmemristor employs lattice scaling [31], ladder scaling [32], and chain scaling [33]. In this section, the frequency-domain characteristic analysis of the recently implemented chain-scaling fracmemristor circuit is considered as an example.

The irregular iterative scaling equation is difficult to solve analytically [31,32,33,37,38]. The operation order of the scaling fracmemristor circuit is approximated, and the solution of the fracmemristance is yet to be solved. In this section, the frequency-domain characteristics of the scaling fracmemristor circuit are analyzed, and the operation order and fracmemristance of the scaling fracmemristor circuit are investigated in the frequency domain. The fracmemristance of the small-signal fracmemristor equivalent circuit is solved in the frequency domain.

First, the configuration and iteration scaling equation of the impedance function of the chain-scaling fracmemristor circuit are presented. Subsequently, the frequency-domain characteristic curves of the reference memristance of the chain-scaling fracmemristor circuit in the maximum and minimum values in the changing range are obtained based on the small-signal impedance function using the order- and F-frequency characteristic functions. Finally, the frequency-domain characteristic curve is analyzed to obtain the effective operating-frequency range of the chain-scaling fracmemristor circuit; the relationship between the fracmemristance and state variable is obtained by fitting.

### 4.1. Chain-Scaling Fracmemristor Circuit

**Figure 3:**
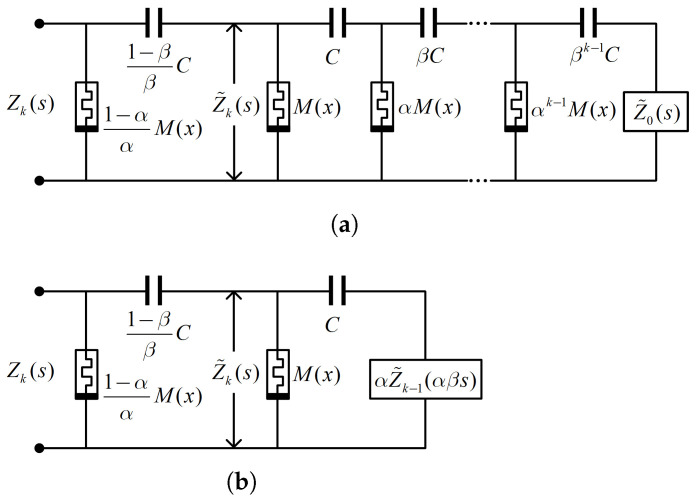
Chain-scaling fracmemristor circuit: (**a**) circuit configuration; (**b**) iterating circuit.

The configuration of the chain-scaling fracmemristor circuit is shown in Figure 3a; and Figure 3b shows the iterating circuit. In these figures, *k* denotes the total number of subcircuits, *C* denotes the reference capacitance, and M(x) denotes the reference memristance. Further, α and β denote the reference memristance and reference capacitance progression ratio, respectively; α and β are positive real numbers, and 0<α,β<1. The chain-scaling fracmemristor circuit is a circuit obtained after the parameter-scaling expansion of the half-order chain-fracmemristor circuit as shown in Figure 1.

According to the iterative circuit shown in Figure 3b, the input impedance ${\tilde{Z}}_{k}\left(s\right)$ is described by the iterative algorithm formula [33,46]

(23) $${\tilde{Z}}_{k}\left(s\right)=\frac{M\left(x\right)\left[1+sC\alpha {\tilde{Z}}_{k-1}\left(\alpha \beta s\right)\right]}{1+sC\alpha {\tilde{Z}}_{k-1}\left(\alpha \beta s\right)+sCM\left(x\right)}.$$

The impedance function ${\tilde{Z}}_{k}\left(s\right)$ shown in Equation (23) belongs to the irregular iterative-scaling equation shown in Equation (3). It is a challenging theoretical problem to obtain the operational order and the fracmemristance from the irregular iterative-scaling equation [31,32,33,37,38]. The operational order approximation of the chain-scaling fracmemristor circuit is provided in Equation (4). The fracmemristor circuit of arbitrary fractional operation order can be obtained by adjusting the values of α and β.

### 4.2. Frequency-Domain Characteristic Analysis of Chain-Scaling Fracmemristor Circuit

**Figure 4:**
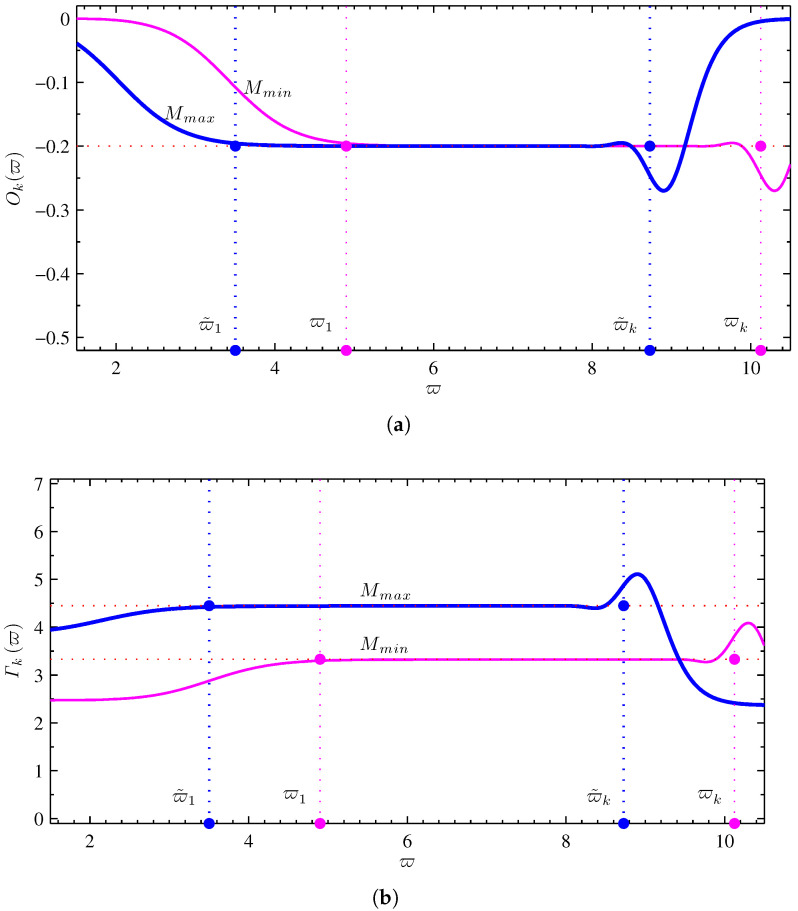
Frequency-domain characteristic curves of the chain-scaling fracmemristor circuit: (**a**) order-frequency characteristic curves; (**b**) F-frequency characteristic curves.

The scaling factor σ=αβ=0.5, k=25, and the reference capacitance C=0.1μF by considering the operation order μ=−0.2 chain-scaling fracmemristor circuit as an example. According to Equation (4), the progressive ratio of the reference memristance α=0.8706 and that of the reference capacitance β=0.5743. In the operating point $Q\left(U_{0},I_{0}\right)$, the state variable $x=X_{0}$, and the small-signal impedance function is ${Z\left(s,Q\right)|}_{M(X_{0})}$. By substituting ${Z\left(s,Q\right)|}_{M(X_{0})}$ into Equations (8) and (9), the order- and F-frequency characteristic curves of the chain-scaling fracmemristor circuit are obtained as shown in Figure 4. Figure 4 shows that $M\left(X_{0}\right)=M_{min}=2$ kΩ and $M\left(X_{0}\right)=M_{max}=50$ kΩ.

According to the order-frequency characteristic curve shown in Figure 4a, the chain-scaling fracmemristor circuit can indeed achieve the operation of order μ=−0.2. The effective operating-frequency range to realize the operation order μ=−0.2 is $\left[\varpi_{1},\varpi_{k}\right]$ and $\left[{\tilde{\varpi }}_{1},{\tilde{\varpi }}_{k}\right]$ when memristance $M\left(X_{0}\right)=M_{min}$ and $M\left(X_{0}\right)=M_{max}$, respectively. The effective operating-frequency range of the operation order μ=−0.2 is changed with the memristance $M(X_{0})$; the frequency range of the overlap $\left[\varpi_{1},{\tilde{\varpi }}_{k}\right]$ is the effective operating-frequency range of the chain-scaling fracmemristor circuit.

**Figure 5:**
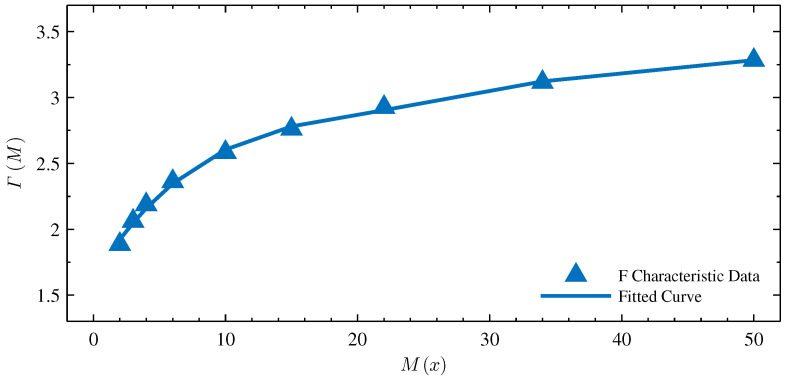
Fitting results of fracmemristance for the chain-scaling fracmemristor circuit.

The F-frequency characteristic curve shown in Figure 4b indicates that the effective operating-frequency range of the operation order μ=−0.2 is the same as the effective operating-frequency range of the fracmemristance. When memristance $M\left(X_{0}\right)=M_{min}$, the effective operating-frequency range to realize the fracmemristance is $\left[\varpi_{1},\varpi_{k}\right]$. When memristance $M\left(X_{0}\right)=M_{max}$, the effective operating-frequency range to realize the fracmemristance is $\left[{\tilde{\varpi }}_{1},{\tilde{\varpi }}_{k}\right]$. There is no analytical or approximate solution for the fracmemristance of the chain-scaling fracmemristor circuit. Within the corresponding effective operating-frequency range of the operating point $Q\left(U_{0},I_{0}\right)$, the average value of the F-frequency characteristic curve function is solved and used as its corresponding fracmemristance. When memristance $M(X_{0})$ from $M_{min}$ changes to $M_{max}$, the change curve of its fracmemristance is as shown in Figure 5.

The relationship curve between the memristance $M(X_{0})$ and F-frequency characteristic curve value shown in Figure 5 is fitted by the least squares method. The resulting relation is

(24) $$\Gamma \left(M\right)=-0.0031M^{2}+0.1078M^{1}+1.7613.$$

Figure 5 also shows the fitted curve; the standard deviation between the error of the fitted curve and data is 0.0453. The formula shown in Equation (24) is called the state dynamic route [14]. Equation (24) indicates that the fracmemristance of the scaling fracmemristor circuit can be solved using the frequency-domain characteristic-analysis method.

## 5. Application of the Frequency-Domain Characteristic-Analysis Principle to Memristors, Memcapacitors, and Meminductors

When the operation orders of the fracmemristor are 0, −1, and +1, it is a memristor, memcapacitor, and meminductor, respectively. The fracmemristor is used as the frequency-domain characteristic-analysis principle of mem-elements to prove its frequency-domain characteristic-analysis principle.

Assuming that the operating point of the memristor is $Q\left(U_{0},I_{0}\right)$, the corresponding state variable $x=X_{0}$ and R(s)=r in Equations (1) and (2). Let the small-signal impedance function be represented by $Z\left(s,Q\right)=FM_{0}{\left(s,Q\right)|}_{R(s)=r}$. According to Equation (8), the order-frequency characteristic function value can then be obtained as

(25) $$O\left(\varpi ,Q\right)=\frac{\mathrm{lg}\left|FM_{0}\left(j2\pi 10^{\varpi },Q\right)\right|}{d\varpi }=0.$$

According to Equation (9), the F-frequency characteristic function value can be obtained as

(26) $$\Gamma \left(\varpi ,Q\right)=\mathrm{lg}F\left(\varpi ,Q\right)=\mathrm{lg}\left|FM_{0}\left(j2\pi 10^{\varpi },Q\right)\right|-O\left(\varpi \right)\left[\varpi +\mathrm{lg}\left(2\pi \right)\right]=\mathrm{lg}r.$$

According to Equations (25) and (26), the order-frequency characteristic function value is equal to the operation order of the memristor; the F-frequency characteristic function value F(ϖ,Q) is equal to the memristance. Thus, it is proved theoretically that the order- and F-frequency characteristic functions are suitable for the frequency-domain analysis of the memristor.

Assuming that the operating point of the memcapacitor is $Q\left(U_{0},I_{0}\right)$, the corresponding state variable $x=X_{0}$ and R(s)=r in Equations (1) and (2). Let the small-signal impedance function be represented by $Z\left(s,Q\right)=FM_{-1}{\left(s,Q\right)|}_{R(s)=r}$. According to Equation (8), the order-frequency characteristic function value can then be obtained as

(27) $$O\left(\varpi ,Q\right)=\frac{\mathrm{lg}\left|FM_{-1}\left(j2\pi 10^{\varpi },Q\right)\right|}{d\varpi }=-1.$$

According to Equation (9), the F-frequency characteristic function value can be obtained as

(28) $$\Gamma \left(\varpi ,Q\right)=\mathrm{lg}F\left(\varpi ,Q\right)=\mathrm{lg}\left|FM_{-1}\left(j2\pi 10^{\varpi },Q\right)\right|-O\left(\varpi \right)\left[\varpi +\mathrm{lg}\left(2\pi \right)\right]=\mathrm{lg}\left(r/c\right).$$

According to Equations (27) and (28), the order-frequency characteristic function value is equal to the operation order of the memristor; the F-frequency characteristic function value F(ϖ,Q) is equal to the inverse of the memcapacitance. Thus, it is theoretically proved that the order- and F-frequency characteristic functions are suitable for the frequency-domain analysis of the memcapacitor.

Assuming that the operating point of the meminductor is $Q\left(U_{0},I_{0}\right)$, the corresponding state variable $x=X_{0}$ and R(s)=r in Equations (1) and (2). Let the small-signal impedance function be represented by $Z\left(s,Q\right)=FM_{+1}{\left(s,Q\right)|}_{R(s)=r}$. According to Equation (8), the order-frequency characteristic function value can be obtained as

(29) $$O\left(\varpi ,Q\right)=\frac{\mathrm{lg}\left|FM_{+1}\left(j2\pi 10^{\varpi },Q\right)\right|}{d\varpi }=1,$$

According to Equation (9), the F-frequency characteristic function value can be obtained as

(30) $$\Gamma \left(\varpi ,Q\right)=\mathrm{lg}F\left(\varpi ,Q\right)=\mathrm{lg}\left|FM_{+1}\left(j2\pi 10^{\varpi },Q\right)\right|-O\left(\varpi \right)\left[\varpi +\mathrm{lg}\left(2\pi \right)\right]=\mathrm{lg}\left(lr\right).$$

According to Equations (29) and (30), the order-frequency characteristic function value is equal to the operation order of the meminductor; further, the F-frequency characteristic function value F(ϖ,Q) is equal to the meminductance. Thus, it is proved theoretically that the order- and F-frequency characteristic functions are suitable for the frequency-domain analysis of the meminductor.

## 6. Conclusions

The frequency-domain characteristic-analysis principle of the fracmemristor was obtained using the order- and F-frequency characteristic functions. The principle of the frequency-domain characteristic analysis was verified by the small-signal impedance function of the ideal fracmemristor. The correctness of the frequency-domain characteristic-analysis principle was also verified by the proposed half-order chain-type fracmemristor circuit from the perspective of circuit configuration, theory, and function curve. The results of this study indicated that the principle of the frequency-domain characteristic analysis of the fracmemristor can successfully calculate the fracmemristance of the chain-scaling fracmemristor circuit.

The implementation process for the frequency domain characteristic analysis of fractional memristor circuit can be summarized as follows: (a) Obtain the small-signal impedance function of the fracmemristor circuit at operating point $Q\left(U_{0},I_{0}\right)$; (b) The specific order- and F-frequency characteristic function are obtained according to the small-signal impedance function; (c) The theoretical settlement results of order- and F-frequency characteristic function can be obtained by substituting parameters, or the curve of order- and F-frequency characteristic function can be drawn; (d) Further analysis was conducted based on the results.

The limitation of this study is that the principle of frequency-domain characteristic analysis, which is based on small-signal analysis, is only applicable to theoretical analysis in most cases. To test the frequency-domain characteristics, the test signal needs to be a small signal. Small signals cannot affect the state variable *x* or ignore the effect on the state variable *x* [47].

The principle of the frequency-domain characteristic analysis employed in this study can be applied to the frequency-domain characteristic analysis of mem-elements, such as the memristor, memcapacitor, and meminductor. The small-signal equivalent circuit of the scaling fracmemristor circuit is the scaling fractor circuit. According to the frequency-domain characteristics analysis principle of the fractor circuit [38,39], the relative error, approximation accuracy, approximation bandwidth, approximation bandwidth exponent, K-diagram, F-index, and approximation benefit of the order- and F-frequency characteristics of the scaling fracmemristor circuit can also be obtained.

## Figures and Tables

**Table 1:** Equations (1) and (2) are the types of elements that can be represented.

Element	μ	η	*p*	${\mathrm{FM}}_{\mu }$	$m_{\mu }\left(x\right)$	R(s)
memcapacitor	−1	1	0	$c^{-1}R\left(s\right)s^{-1}$	$c^{-1}L^{-1}\left\{R(s)\right\}$	R(s)
capacitive fracmemristor	−1<μ<0	0	−μ	$c^{\mu }{\left[R\left(s\right)\right]}^{1+\mu }s^{\mu }$	$c^{\mu }L^{-1}\left\{{\left[R\left(s\right)\right]}^{1+\mu }\right\}$	
memristor	0	0	0	R(s)	$L^{-1}\left\{R(s)\right\}$	
inductive fracmemristor	0<μ<1	0	μ	$l^{\mu }{\left[R\left(s\right)\right]}^{1-\mu }s^{\mu }$	$l^{\mu }L^{-1}\left\{{\left[R\left(s\right)\right]}^{1-\mu }\right\}$	
meminductor	1	1	0	lR(s)s	$lL^{-1}\left\{R(s)\right\}$		
capacitor	−1	1	0	$c^{-1}rs^{-1}$	$c^{-1}r$	*r*
capacitive fractor	−1<μ<0	0	−μ	$c^{\mu }r^{1+\mu }s^{\mu }$	$c^{\mu }r^{1+\mu }$	
resistor	0	0	0	*r*	*r*	
inductive fractor	0<μ<1	0	μ	$l^{\mu }r^{1-\mu }s^{\mu }$	$l^{\mu }r^{1-\mu }$	
inductor	1	1	0	lrs	lr		

## Data Availability

Not applicable.
